# Hospital-based surveillance of severe paediatric malaria in two malaria transmission ecological zones of Burkina Faso

**DOI:** 10.1186/s12936-022-04433-x

**Published:** 2023-01-06

**Authors:** Alfred B. Tiono, Amadou T. Konaté, Désiré Kargougou, Amidou Diarra, Issa Nébié Ouedraogo, Amidou Ouedraogo, Franco Pagnoni, David Modiano, Sodiomon B. Sirima

**Affiliations:** 1 Groupe de Recherche Action en Santé (GRAS), 06 BP, 1028 Ouagadougou, Burkina Faso; 2 Centre National de Recherche et de Formation sur le Paludisme (CNRFP), 01 BP, 2208 Ouagadougou, Burkina Faso; 3 The Barcelona Institute for Global Health, Rosselló, 132, 7è08036 Barcelona, Spain; 4 University “La Sapienza” of Roma, Roma, Italy

**Keywords:** Severe malaria, Admission, Children, Transmission zone, Burkina Faso

## Abstract

**Background:**

In the current context of tailoring interventions to maximize impact, it is important that current data of clinical epidemiology guide public health programmes and health workers in the management of severe disease. This study aimed at describing the burden of severe malaria at hospital level in two areas with distinct malaria transmission intensity.

**Methods:**

A hospital-based surveillance was established in two regional hospitals located in two areas exposed to different malaria transmission. Data on paediatric severe malaria admissions were recorded using standardized methods from August 2017 to August 2018 with an interruption during the dry season from April to June 2018.

**Results:**

In total, 921 children with severe malaria cases were enrolled in the study. The mean age was 33.9 (± 1.3) and 36.8 (± 1.6) months in lower malaria transmission (LMT) and higher malaria transmission (HMT) areas (p = 0.15), respectively. The geometric mean of asexual *P. falciparum* density was significantly higher in the LMT area compared to the HMT area: 22,861 trophozoites/µL (95% CI 17,009.2–30,726.8) vs 11,291.9 trophozoites/µL (95% CI 8577.9–14,864.5). Among enrolled cases, coma was present in 70 (9.2%) participants. 196 patients (21.8%) presented with two or more convulsions episodes prior to admission. Severe anaemia was present in 448 children (49.2%). Other clinical features recorded included 184 (19.9%) cases of lethargy, 99 (10.7%) children with incoercible vomiting, 80 (8.9%) patients with haemoglobinuria, 43 (4.8%) children with severe hypoglycaemia, 37 (4.0%) cases where child was unable to drink/suck, 11 (1.2%) cases of patients with circulatory collapse/shock, and 8 cases (0.9%) of abnormal bleeding (epistaxis). The adjusted odds of presenting with coma, respiratory distress, haemoglobinuria, circulatory collapse/shock and hypoglycaemia were significantly higher (respectively 6.5 (95%CI 3.4–12.1); 1.8 (95%CI 1.0–3.2); 2.7 (95%CI 1.6–4.3); 5.9 (95%CI 1.3–27.9); 1.9 (95%CI 1.0–3.6)) in children living in the HMT area compared to those residing in the LMT area. Overall, forty-four children died during hospitalization (case fatality rate 5.0%) with the highest fatalities in children admitted with respiratory distress (26.0%) and those with hypoglycaemia (25.0%).

**Conclusion:**

The study showed that children in the HMT area have a higher risk of presenting with coma, shock/dehydration, haemoglobinuria, hypoglycaemia, and respiratory distress. Case-fatality rate is higher among patients with respiratory distress or hypoglycaemia. Hospital surveillance provides a reliable and sustainable means to monitor the clinical presentation of severe malaria and tailor the training needs and resources allocation for case management.

## Background

Despite the recent progress in malaria control in many countries across the globe, malaria remains the main public health threat in sub-Saharan Africa. Globally, there were an estimated 229 million malaria cases in 2019 in 87 malaria endemic countries, with about 94% of cases accounted for in the World Health Organization (WHO) African Region [1].

Burkina Faso is one of those countries where progress in controlling the disease has stalled despite full-scale deployment of available interventions which include case management using artemisinin-based combination therapy (ACT), vector control through large-scale distribution of long-lasting insecticidal nets (LLINs); and seasonal malaria chemoprevention (SMC) targeting the marked seasonality of the disease transmission. The country is one of the high-burden nations as defined by the WHO and is part of the high burden to high impact initiative [2].

The clinical epidemiology of severe, life-threatening malaria was characterised in Burkina Faso during the 1990s [3] at a time where current control tools were not yet deployed. These clinical features may no longer be relevant todays. Indeed, there is evidence that some interventions such as LLINs had led to a decrease of anaemia among children in Kenya. It is possible that current interventions at scale may have changed the clinical presentation of severe malaria, by impacting on transmission intensity [4, 5].

To tailor interventions to maximize impact, it is important to obtain up to date information on the epidemiology of severe malaria. This study describes the current burden of severe malaria in children admitted at hospital level in two areas with distinct malaria transmission. The information on prevalence and lethality of severe malaria in hospitalized children is useful to the National Malaria Control Programme (NMCP), to health workers and may contribute to fine tuning the malaria control strategies and resources allocation.

## Methods

### Study sites

The study was conducted at two regional referral hospitals, the Regional Hospital of Koudougou and the Regional Hospital of Banfora, respectively. The Regional Hospital of Koudougou (lower transmission area, LMT) is located 100 km Centre West of the capital city of Ouagadougou. Malaria transmission in this central part of the country is markedly seasonal and intense during the rainy season from June to October. The entomological inoculation rate (EIR) was estimated at 31.4 infective bites/person/year in a study conducted in the neighbouring zone of Saponé [6]. The Banfora Regional Hospital (higher transmission area, HMT) is located within a very highly endemic area where transmission occurs throughout the year, with a peak during the rainy season (from June to October). The EIR ranges from 55 to 400 infected bites/person/year (Tiono et al., unpublished data). In both areas, the main malaria vectors are *Anopheles gambiae*, *Anopheles arabiensis* and *Anopheles funestus,* and *Plasmodium falciparum* is responsible for more than 90% of all clinical malaria cases [7, 8].

### Recruitment and management of study participants

The recruitment of children took place from August 2017 to August 2018 with an interruption from April to June 2018 (representing the dry season). For all consecutive children aged 0 to 15 years presenting at the paediatric emergency room at any time with a clinical presentation suggestive of severe malaria, an informed consent was obtained by study personnel from parents/legal representatives. Patients were further assessed by paediatric ward clinicians (who benefited from a refresher protocol specific training prior to the study start) and observations recorded using a standardized case report form. Data collected included the medical history, vital signs, a complete physical examination for signs of severity (affecting the airway, respiratory, circulatory, and neurological systems). Finger prick blood sample was taken for malaria smear (methods for microscopy examination of blood smears and criteria for diagnosis of severe malaria are given below), haemoglobin and glucose measurements using point of care devices (HemoCue® Hb 201 + and HemoCue® Glucose 201 + ; HaemoCue AB, Ängelholm, Sweden). If the RDT was positive, anti-malarial treatment was immediately initiated based on the NMCP guidelines.

Children received parenteral artesunate at 2.4 mg/kg body weight on admission (time zero), then after 12 and 24 h and then once a day, or artemether Injection at 3.2 mg/kg on admission, then 1.6 mg/kg body weight per day. In both cases, parenteral administration continued until the child improved and was able to take full course of oral ACT, using artemether-lumefantrine or artesunate-amodiaquine. Supportive therapy recommended for severe malaria includes the treatment of hypoglycaemia with dextrose when glucose < 2.2 mmol/l, blood transfusion for children with haemoglobin less than 5 g/dL. All treatments were free of charge. No adjusted dose of parenteral artesunate for children weighing less than 20 kg was adopted by the NMCP at the time of the study conduct.

### Blood smears reading

Blood films were managed as described elsewhere [9]. All RDT positive children had blood smears taken and examined by light microscopy. In brief, smears were air-dried and Giemsa-stained for examination by a light microscope fitted with 100 X oil immersion lens at a single laboratory. At least two hundred thick film fields were examined before a slide was declared negative. If *Plasmodium* asexual forms were found, a total of two hundred thick film fields were screened for *Plasmodium* species other than *P. falciparum*. If *P. falciparum* was present, a count of asexual forms against leukocytes was made using a tally counter. Counting was conducted based on at least two hundred leukocytes in consistence with WHO standards. If less than 10 parasites were identified from the two hundred leukocytes screened, the count was extended to 1,000 leukocytes. On the other hand, if *P. falciparum* gametocytes were seen, a gametocyte count was performed against 1,000 leukocytes. All slides were read by two independent microscopists with a third reading in case of discordant results. The final result was based on the two most concordant readings. All readers are certified competent/expert by an external quality assurance system with Clinical Laboratory Services (http://www.cls.co.za), South Africa.

### Severe malaria case definitions

Severe malaria was defined based on presence of one or more criteria outlined in box 1 with a malaria positive RDT result. Only haemoglobin and glucose were measured in study participants. Therefore, it was not possible to assess the incidence of other clinical features as defined by the WHO; among others the hyperlactataemia, hyperbilirubinaemia, metabolic acidosis, renal failure. Passing dark urine was considered as a proxy of haemoglobinuria. As per country NMCP guidelines, the study has included in severe cases definitions children with incoercible vomiting, lethargy, and inability to drink or suck.

Box 1 Features of severe malaria Clinical features of severe malaria.

Impaired consciousness (including unrousable coma);

Prostration, i.e. generalized weakness so that the patient is unable to sit, stand or walk without assistance.

Multiple convulsions: more than two episodes within 24 h;

Acute pulmonary oedema and acute respiratory distress syndrome.

Circulatory collapse or shock, systolic blood pressure.

Clinical jaundice plus evidence of other vital organ dysfunction.

Abnormal bleeding.

Haemoglobinuria (dark urine or coca cola);

Laboratory findings.

Hypoglycaemia (< 2.2 mmol/l or < 40 mg/dl);

Severe anaemia (haemoglobin < 5 g/dl).

Danger signs as per NMCP guidelines.

Incoercible vomiting.

Lethargy.

Inability to drink or suck.

### Statistical methods

Data were entered into a MS ACCESS database and analysed using STATA software (Version 14.0, College Station, TX: StataCorp; 2015). Analysis included all paediatric admissions meeting the study eligibility criteria. Frequency were used for qualitative data and mean or geometric for quantitative data. The Clopper–Pearson method was used for calculating binomial confidence intervals. For computing the case fatality rates (CFR), only individuals with known in hospital outcome were included. Multivariable regression analysis was done to assess association between clinical features and transmission intensity. The significance level was set at P < 0.05.

### Ethical and regulatory approvals

The study protocol and associated documents were reviewed and approved by CNRFP institutional bioethics committee (approval reference N° 2015/007/MS/SG/CNRFP/CNRFP/CIB), the Ministry of Health Ethical Committee for Biomedical Research (approval reference N° 2015–7-092). All study participants’ parents or legal representatives gave documented informed consent before any study procedures were performed. The study was conducted according to the principles of the declaration of Helsinki and International Conference on Harmonization (ICH) Good Clinical Practice (GCP) guidelines.

## Results

### Population characteristics

Characteristics of enrolled children are presented in Table 1. A total of 921 severe malaria cases were admitted based on RDT positivity. Among them, only 448 (48.6%) were confirmed positive by microscopy. Overall, at admission, 46.8% and 39.0% have received an antimalarial treatment pre-referral respectively in the LMT and the HMT area (p = 0.017). Among those who were microscopy negative at admission, 44.6% had received an anti-malarial treatment pre-referral to the hospital. Injectable artesunate was the most administered drug (87.0%), followed by artemether-lumefantrine (AL) (11.5%). Few numbers of cases (2.5%) have received both injectable artesunate then oral AL.

**Table 1:** Characteristics of enrolled children

Characteristics	Lower transmission area	Higher transmission area	Overall
Number enrolled	506 (54.9%)	415 (46.1%)	921 (100%)
Gender			
Male	272 (53.7%)	241 (58.1%)	513 (55.7%)
Female	234 (46.3%)	174 (41.9%)	408 (44.3%)
Mean time of illness before hospital admission in days (95%CI)	3.0 (2.7–3.4)	4.0 (3.5–4.5)	3.5 (3.2–3.8)
Proportion who received anti-malarial treatment before admission (95% CI)	46.8% (42.4–51.3)	39.0% (34.3–43.9)	43.3% (40.0–46.6)
Proportion with fever at admission (95% CI)	79.2% (75.4–82.7)	75.2% (70.7–79.3)	77.4% (75.5–80.0)
Mean age in months (95% CI)			
Coma	48.6 (33.9–64.1)	36.9 (30.9–42.8)	39.0 (33.5–44.6)
Convulsions	29.5 (25.5–33.5)	38.3 (32.1–44.5)	33.7 (30.0–37.3)
Prostration	39.3 (34.1–44.6)	41.9 (37.6–46.2)	40.9 (37.5–44.2)
Respiratory Distress	32.1 (17.2–47.0)	29.3 (16.8–41.9)	30.5 (21.2–39.7)
Abnormal bleeding	66.8 (31.2–102.4)	-	-
Circulatory collapse/shock	9 (6.0–47)	42.4 (18.3–66.6)	36.4 (15.4–57.3)
Haemoglobinuria	50.6 (34.7–66.4)	45.3 (36.1–54.5)	46.9 (39.1–54.8)
Incoercible vomiting	34.9 (28.1–41.8)	39.0 (30.2–47.8)	36.7 (31.3–42.0)
Lethargy	35.1 (30.2–40.0)	47.8 (37.0–58.5)	37.6 (33.1–42.1)
Inability to drink /suck	37.7 (21.9–53.5)	41.7 (27.8–55.5)	39.6 (29.6–49.7)
Severe anaemia	30.9 (27.3–34.5)	31.6 (27.8–35.5)	31.2 (28.6–33.8)
Hypoglycaemia	29.2 (14.5–43.9)	26.2 (19.9–32.6)	27.4 (20.8–34.0)
Proportion of microscopically confirmed *P. falciparum* infection (95% CI)	42.7% (38.3–47.1)	55.9% (50.9–60.7)	48.6% (45.4–51.9)
Geometric mean of *P. falciparum* count (trophozoites/µL) (95% CI)	22,861.3 (17,009.2–30,726.8)	11,291.9 (8,577.9–14,864.5)	15,865.8 (12,946.4–19,443,6)
Ethnicity (95% CI)			
Fulani	4.3% (2.7–6.5)	9.6% (7.0–12.9)	6.7% (5.2–8.5)
Other ethnic groups	95.7% (93.5–97.2)	90.4% (87.1–93.0)	93.3% (91.4–94.8)
Proportion with recent history of malaria/fever episode (current malaria season)	42.5% (38.1–46.9)	70.1% (65.5–74.5)	54.9% (51.7–58.2)

The mean time of illness before hospital admission was 3.0 days (95% CI 2.7–3.4) and 4.0 days (95% CI 3.5–4.5) in the LMT and the HMT areas, respectively. A high proportion of children admitted (38.4%) had also received a beta lactam antibiotic prior to hospital admission. There was a statistically significant difference between the HMT and LMT areas (25.6% vs 34.4% respectively; p = 0.01). Antibiotics administered include ceftriaxone (59.2%); ampicillin (29.6%) and amoxicillin (8.5%). Overall, almost half (43.1%) of children who had received an antimalarial were also given an antibiotic.

The age of recruited children was 33.9 (± 1.3) and 36.8 (± 1.6) months in respectively LMT and HMT areas (p = 0.15)). For children enrolled with convulsions, the mean age was 29.5 months in LMT area compared to 38.3 in the HMT area. The difference was statistically significant.

The geometric mean of asexual *P. falciparum* density was significantly higher in the LMT area compared to the HMT area (22,861 trophozoites/µL;95% 17,009.2–30,726.8 vs 11,291.9 trophozoites/µL; 95% CI 8577.9–14,864.5). In total 58 (6.3%) of enrolled cases were harbouring *P. falciparum* gametocytes. The proportion of patients with gametocytaemia were 4.9% and 7.9% in LMT and HMT areas (p = 0.06), respectively. The geometric mean of gametocytes density was 44.7 gametocytes/µL (95% CI 25.4–78.7) and 81.3 gametocytes/µL (95%CI 52.2–126.5) in the LMT and HMT areas, respectively.

### Clinical presentation of severe malaria cases

**Fig. 1:**
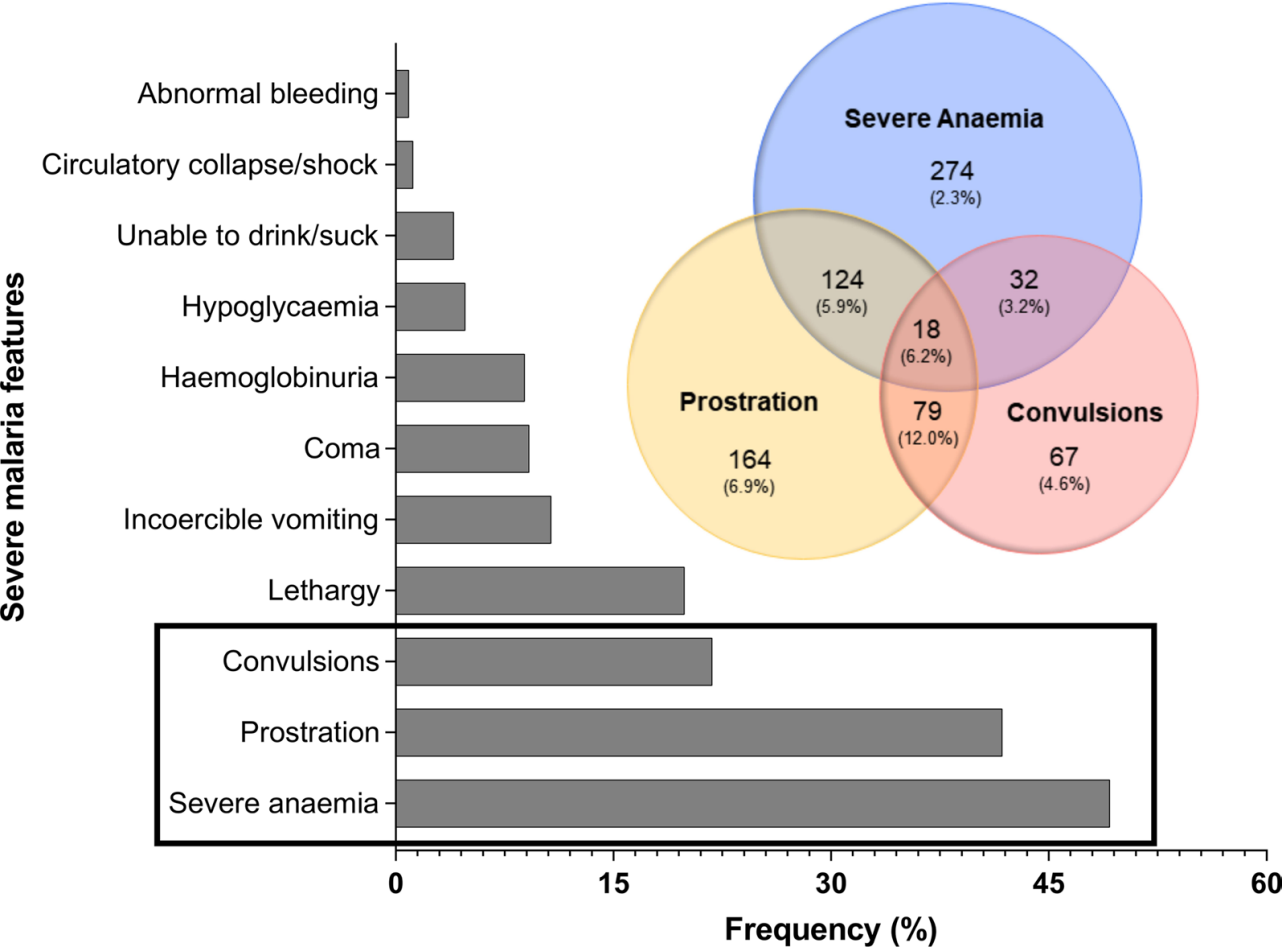
Clinical presentation and overlap between the top three predominant severe syndromes. The percentage case fatality rate are in brackets

Clinical presentation and overlap between the top three predominant syndromes are presented in Fig. 1. Among enrolled cases, coma was present in 70 (9.2%) participants. There were 291 children presenting convulsions prior to admissions. The number of convulsive episodes ranged from 1 to 10. Two or more convulsions prior to admissions were recorded in 196 patients (21.8%).

The mean haemoglobin level was 5.7 (± 2.7) g/dL in study participants. Severe anaemia was present in 448 children (49.2%). On admission, other clinical features recorded included 8 cases (0.9%) of abnormal bleeding (epistaxis), 11 (1.2%) cases of circulatory collapse/shock, 80 (8.9%) patients with haemoglobinuria, 99 (10.7%) children with incoercible vomiting, 184 (19.9%) cases of lethargy, 37 (4.0%) children unable to drink/suck and 385 (41.8%) children with prostration. Among those with haemoglobinuria, only 7 (13.9%) have reported Quinine intake during the episode. The mean glucose level was 5.8 mmol/l (± 2.8). Severe hypoglycaemia was present in 43 (4.8%) children. The proportions of children presenting with one (01), two (02), three or more (≥ 3) severe features were 48.8%, 33.2% and 18.0%, respectively.

### Clinical presentation of severe malaria clinical in the two study sites

Children living in the HMT area tend to present with three or more severe features compared to those from the LMT area (57.8% vs. 42.2%, p = 0.001). The association between clinical presentation and malaria endemicity is presented in Table 2. The odds of presenting with coma, prostration, respiratory distress, haemoglobinuria, circulatory collapse/shock, and hypoglycaemia were significantly higher in children living in the HMT area compared to those residing in the LMT area. The odds remains higher after adjusting for antimalarial treatment (pre-referral) and parasite density on admission.

**Table 2:** Association between clinical presentation and malaria endemicity

				Odd ratio (OR)	
Clinical presentation	Total	Lower transmission area	Higher transmission area	Unadjusted OR (with 95% CI)	Adjusted OR (with 95% CI)
Coma	70	13 (2.6%)	57 (13.7%)	6.0 (3.2–11.2)**	6.5 (3.4–12.1) **
Convulsions	196	105 (20.8%)	91 (21.9%)	1.0 (0.8–1.5)	1.1 (0.8–1.5)
Prostration	385	162 (32.0%)	223 (53.7%)	2.5 (1.9–3.2) **	2.5 (1.9–3.3) **
Respiratory Distress	53	22 (4.3%)	31 (7.5%)	1.8 (1.0–3.1) **	1.8 (1.0–3.2) **
Abnormal bleeding	8	7 (1.4%)	1 (0.2%)	0.2 (0.02–1.4)	0.2 (0.1–1.3)
Circulatory collapse/shock	11	2 (0.4%)	9 (2.2%)	5.6 (1.2–26.0) **	5.9 (1.3–27.9) **
Haemoglobinuria	80	26 (5.1%)	54 (13.0%)	2.8 (1.7–4.5) **	2.7 (1.6–4.3) **
Incoercible vomiting	99	57 (11.2%)	42 (10.1%)	0.9 (0.6–1.4)	0.9 (0.6–1.3)
Lethargy	184	149 (29.4%)	35 (8.4%)	0.2 (0.1–0.3)	0.2 (0.1–0.3)
inability to drink /suck	37	19 (3.7%)	18 (4.3%)	1.2 (0.6–2.2)	1.1 (0.6–2.2)
Severe anaemia	448	252 (50.7%)	196 (47.5%)	0.9 (0.7–1.1)	0.9 (0.7–1.1)
Hypoglycaemia	43	17 (3.4%)	26 (6.4%)	1.9 (1.0–3.6) **	1.9 (1.0–3.6) **

^**^statistically significant difference between LMT and HMT

### Case fatality in study participants with various severe malaria clinical features

Table 3 summarizes the case fatality ratio for each clinical presentation of severe malaria. Overall, forty-four children died during hospitalization (case fatality rate (CFR) 4.8%). Among them, 11 (25%) were microscopically negative at admission. In total 80% of death were recorded in children younger than 5 years. 81.8% of deaths occurred within the first 2 days of admission and 80% of death occurred in children less than five. The CFR was proportional to the number of clinical features at admission. It was 2.3%, 4.6% and 12.1% in children presenting with one, two and three or more clinical features, respectively. The difference was statistically significant. Overall, CFR were highest among children with respiratory distress (26.0%), hypoglycaemia (25.0%); circulatory collapse/shock (18.8%) and those with inability to drink/suck (19.4%). The coma was also associated with a high CFR (16.7%) and the CFR for children with two or more convulsions at admission was 7.4%. The CFR was much lower for children with prostration (7.6%), haemoglobinuria (5.3%), incoercible vomiting (4.1%), lethargy (4.5%) and anaemia (3.6%). No death was recorded in children with abnormal bleeding at admission.

**Table 3:** Case fatality rates associated with the different clinical presentations

	CFR (%, 95%CI)		
Clinical presentation	Lower transmission area	Higher transmission area	Overall
Coma	2/13 (15.3%, 1.9–45.4%)	9/53 (16.9%, 8.1–29.8%))	11/66 (16.7%, 8.6–27.9%))
Convulsions	7/102 (6.9%, 2.8–13.6%))	7/86 (8.1%, 3.3–16.0%)	14/188 (7.4%, 4.1–12.2%)
Prostration	10/154 (6.5%, 3.1–11.6%)	18/213 (5.1–13.0%)	28/367 (7.6%, 5.1–10.8%)
Respiratory Distress	5/21 (23.8%, 8.2–47.2%)	8/29 (27.6%, 12.8–47.2%)	13/50 (26.0%, 14.6–40.3%)
Abnormal bleeding	–	–	–
Circulatory collapse/shock	0/2 (–)	2/9 (22.2%, 2.8–60.0%)	2/11 (18.2%, 2.3–51.8%)
Haemoglobinuria	1/23 (4.3%, 0.1–21.9%))	3/53 (5.7%, 1.2–15.7%)	4/76 (5.3%, 1.4–12.9%)
Incoercible vomiting	3/56 (5.4%, 1.1–14.9%)	1/42 (2.4%, 0.1–12.6%)	4/98 (4.1%, 1.1–10.1%)
Lethargy	5/144 (3.5%, 1.1–7.9%)	3/34 (8.8%, 1.8–23.7%)	8/178 (4.5%, 1.9–8.7%)
Inability to drink /suck	5/18 (27.8%, 9.7–53.5%)	2/18 (11.1%, 1.4–34.7%)	7/36 (19.4%, 8.2–36.0%)
Severe anaemia	7/243 (2.9%, 1.2–5.8%)	8/180 (4.4%, 1.9–8.6%)	15/423 (3.5%, 2.0–5.8%)
Hypoglycaemia	3/17 (17.6%, 3.8–43.4%)	7/23 (30.4%, 13.2–52.9%)	10/40 (25%, 12.7–41.2%)

## Discussion

This study investigated the burden of severe malaria in two hospitals located in two areas with different malaria transmission covered by same malaria control strategies as deployed the country NMCP.

The predominant clinical presentations were anaemia (49.2%) and convulsions (21.8%); almost at the same level as reported in Burkina more than 20 years before by Modiano et al. [10]. This is unexpected given the context of seasonal malaria chemoprevention implementation (SMC) and the high coverage reported [11].

SMC consists of 4 rounds of administration of sulfadoxine/pyrimethamine + Amodiaquine given at intervals of one month to children aged between 3 and 59 months during the peak transmission season (July to October). This strategy has been shown to drastically reduce incidence of uncomplicated and severe malaria due to *P. falciparum* infection and prevalence of malaria-related anaemia [12–14] in children who benefit. However, only the first dose of the three-day treatment regimen is given under direct observation. The prevalence of severe anaemia was two time higher than what was reported in Kenya and Mozambique. The same studies reported a lower prevalence of convulsions (13.6%) and 8.9% respectively [15, 16]. In total 4.0% of enrolled was unable to drink/suck compared to 29.7% reported in previous study [15]. The prevalence of prostration and circulatory collapse/shock was lower compared to previous findings; while it was comparable for hypoglycaemia [16]. The proportions of children presenting with only one 48.8% in the study compared to 78.4% in Mozambique [16].

The odds of presenting with coma was significantly higher in children living in the higher malaria transmission area compared to those in lower malaria transmission area. This finding is in line with the inversely correlated geometric mean parasite. These findings corroborate previous report from Uganda [17], but are not in line with previous studies conducted in sub‐Saharan Africa, which have shown that cerebral malaria (CM) is mostly predominant in low transmission intensity area as compared to high transmission areas [10, 18, 19]. It was hypothesized that the level of acquired immunity is an important determinant of susceptibility to CM [20]. It is also possible that the SMC which maintains children under prophylaxis during the high transmission season altered the naturally acquired, *P. falciparum* erythrocyte membrane protein 1 (PfEMP1) specific immunity which has been described to play a significant role in the susceptibility to CM [21].

Shock/dehydration was the second clinical presentation more observed in HMT area compared to LMT area (adjusted OR = 5.3 (1.1–24.6)). There are many potential causes of hypotension or shock in severe malaria. These include secondary bacterial sepsis, dehydration from vomiting or fever, haemorrhage from gastrointestinal bleeding, splenic rupture, and cardiac dysfunction. This was an unexpected finding which calls for further investigations to understand the potential causes of the difference if confirmed.

Haemoglobinuria was one of the clinical syndromes significantly associated with the transmission intensity. The odds of presenting with haemoglobinuria was almost 3 times in HMT area although the overall incidence was similar to what was reported elsewhere in west Africa [22]. A number of factors including the immune hypersensitivity in long-term residents of *P. falciparum*-endemic areas who took quinine, the glucose-6-phosphate dehydrogenase (G6PD) deficiency have been described as potential causes of haemoglobinuria. Occurrence of haemoglobinuria have also been described in patients with normal erythrocyte G6PD activity who were administered quinine for severe malaria [23, 24]. However, in the present study, all patients who report quinine intake were recorded in the LMT area. In previous, work the prevalence of G6PD deficiency by enzymatic test was higher in LMT area compared the HMT area [25]. Another trigger is an antibiotic induced haemolysis. Indeed, ceftriaxone has been found to induce haemolysis in many reports [26, 27]. In the current study, it was the most prescribed antibiotic (59.2%), although, predominantly in the LMT area (34.4%vs 25.6%).

The overall CFR recorded in this study is relatively high (5.0%) but remains within findings reported elsewhere [15–17, 28]. It was however, reduced by half compared to previous reports in Burkina Faso [10]. This may be due to the improved quality of care during these last 20 years in the country. Indeed beside the use of artemisinin derivatives in the treatment of severe malaria, it was also noticed that a high proportion of study participants have received pre referral anti-malarial (43.3%) and an antibiotic (38.4%) treatment. CFR was the highest in children with respiratory distress (26.0%) and those presenting with hypoglycaemia (25%). CFR was also high in children with shock/severe dehydration. In this study it was not possible to perform blood culture and initiate adequate antibiotics treatment. It is possible that bacterial co-infections may have been overlooked. Although most predominant, the CFR associated with severe malaria anaemia was among the lowest, in line with what previously observed by Marsh et al.[29].

More than half (51.4%) of cases referred by peripheral health clinics, who were admitted and managed as severe malaria cases based on HRP2 RDT results turned out to be negative by microscopy probably due to the high proportion (44.6%) among them who had received pre-referral anti-malarial treatment or alternatively to the low specificity of the HRP2 based RDT in our geographical context [9, 30, 31]. The high proportion of microscopically negative cases may have potential deleterious consequences for the appropriate management of hospitalized children. It is indeed possible that these children with false-positive RDT are suffering from other underlying causes to their conditions which were undetected given the focus of the clinical team on severe malaria [18]. The training of the health staff at the tertiary level of the health system on these risks of mismanagement need to be emphasized; so that appropriate complementary investigations are undertaken whenever needed.

## Conclusion

In two areas of Burkina Faso with different ecology of malaria transmission, hospital-based surveillance of severe malaria clinical presentations has shown that children in the HMT are more significantly at risk of presenting with coma, prostration, shock/dehydration, haemoglobinuria, hypoglycaemia, and respiratory distress. Case-fatality rates are high among patients with respiratory distress and those with hypoglycaemia. Most of the deaths have occurred in children aged less than five years. Hospital surveillance provides a reliable and sustainable means to monitor the clinical presentations and lethality of severe and tailor the training needs for health workers at the frontline of cases management in different transmission patterns.

## Data Availability

The datasets analysed during the current study are available from the corresponding author on reasonable request.

## Abbreviations

ACT: Artemisinin-based combination therapy
CFR: Case fatality rate
CM: Cerebral malaria
EIR: Entomological inoculation rate
G6PD: Glucose-6-phosphate dehydrogenase
HMT: Higher malaria transmission area
HRP2: Histidine Rich Protein 2
LLIN: Long-lasting insecticidal net
LMT: Lower malaria transmission area
RDT: Rapid diagnosis test
SMC: Seasonal malaria chemoprevention
WHO: World Health Organization

## References

[1] WHO. World Malaria Report 2020. Geneva, World Health Organization, 2020. https://www.whoint/docs/default-source/malaria/world-malaria-reports/9789240015791-double-page-viewpdf?sfvrsn=2c24349d_5.

[2] WHO. High Burden to high impact-a targeted malaria response. Geneva, World Health Organization, 2018. https://apps.whoint/iris/bitstream/handle/10665/275868/WHO-CDS-GMP-201825-engpdf.

[3] Modiano D, Sirima BS, Sawadogo A, Sanou I, Pare J, Konate A (1999). Severe malaria in Burkina Faso: urban and rural environment. Parassitologia.

[4] Frosch AE, Ondigo BN, Ayodo GA, Vulule JM, John CC, Cusick SE (2014). Decline in childhood iron deficiency after interruption of malaria transmission in highland Kenya. Am J Clin Nutr.

[5] Gitonga CW, Edwards T, Karanja PN, Noor AM, Snow RW, Brooker SJ (2012). *Plasmodium* infection, anaemia and mosquito net use among school children across different settings in Kenya. Trop Med Int Health.

[6] Tiono AB, Guelbeogo MW, Sagnon NF, Nebie I, Sirima SB, Mukhopadhyay A (2013). Dynamics of malaria transmission and susceptibility to clinical malaria episodes following treatment of *Plasmodium falciparum* asymptomatic carriers: results of a cluster-randomized study of community-wide screening and treatment, and a parallel entomology study. BMC Infect Dis.

[7] Kangoye DT, Nebie I, Yaro JB, Debe S, Traore S, Ouedraogo O (2014). *Plasmodium falciparum* malaria in children aged 0–2 years: the role of foetal haemoglobin and maternal antibodies to two asexual malaria vaccine candidates (MSP3 and GLURP). PLoS ONE.

[8] Tiono AB, Kangoye DT, Rehman AM, Kargougou DG, Kabore Y, Diarra A (2014). Malaria incidence in children in South-West Burkina Faso: comparison of active and passive case detection methods. PLoS ONE.

[9] Tiono AB, Ouedraogo A, Diarra A, Coulibaly S, Soulama I, Konate AT (2014). Lessons learned from the use of HRP-2 based rapid diagnostic test in community-wide screening and treatment of asymptomatic carriers of *Plasmodium falciparum* in Burkina Faso. Malar J.

[10] Modiano D, Sirima BS, Sawadogo A, Sanou I, Pare J, Konate A (1998). Severe malaria in Burkina Faso: influence of age and transmission level on clinical presentation. Am J Trop Med Hyg.

[11] Partnership A-SMC (2020). Effectiveness of seasonal malaria chemoprevention at scale in west and central Africa: an observational study. Lancet.

[12] Diawara F, Steinhardt LC, Mahamar A, Traore T, Kone DT, Diawara H (2017). Measuring the impact of seasonal malaria chemoprevention as part of routine malaria control in Kita. Mali Malar J.

[13] Druetz T (2018). Evaluation of direct and indirect effects of seasonal malaria chemoprevention in Mali. Sci Rep.

[14] Kweku M, Liu D, Adjuik M, Binka F, Seidu M, Greenwood B (2008). Seasonal intermittent preventive treatment for the prevention of anaemia and malaria in Ghanaian children: a randomized, placebo controlled trial. PLoS ONE.

[15] Akech S, Chepkirui M, Ogero M, Agweyu A, Irimu G, English M (2020). The clinical profile of severe pediatric malaria in an area targeted for routine RTS, S/AS01 malaria vaccination in Western Kenya. Clin Infect Dis.

[16] Bassat Q, Guinovart C, Sigauque B, Aide P, Sacarlal J, Nhampossa T (2008). Malaria in rural Mozambique. Part II: children admitted to hospital. Malar J.

[17] Idro R, Aloyo J, Mayende L, Bitarakwate E, John CC, Kivumbi GW (2006). Severe malaria in children in areas with low, moderate and high transmission intensity in Uganda. Trop Med Int Health.

[18] Reyburn H, Mbatia R, Drakeley C, Bruce J, Carneiro I, Olomi R (2005). Association of transmission intensity and age with clinical manifestations and case fatality of severe *Plasmodium falciparum* malaria. JAMA.

[19] Roca-Feltrer A, Carneiro I, Smith L, Schellenberg JR, Greenwood B, Schellenberg D (2010). The age patterns of severe malaria syndromes in sub-Saharan Africa across a range of transmission intensities and seasonality settings. Malar J.

[20] Postels DG, Birbeck GL (2013). Cerebral malaria. Handb Clin Neurol.

[21] Jensen AR, Adams Y, Hviid L (2020). Cerebral *Plasmodium falciparum* malaria: the role of PfEMP1 in its pathogenesis and immunity, and PfEMP1-based vaccines to prevent it. Immunol Rev.

[22] Ajetunmobi WA, Orimadegun AE, Brown BJ, Afolabi NK, Olabiyi FA, Anetor JI (2012). Haemoglobinuria among children with severe malaria attending tertiary care in Ibadan. Nigeria Malar J.

[23] Sodeinde O (1992). Glucose-6-phosphate dehydrogenase deficiency. Baillieres Clin Haematol.

[24] Tran TH, Day NP, Ly VC, Nguyen TH, Pham PL, Nguyen HP (1996). Blackwater fever in southern Vietnam: a prospective descriptive study of 50 cases. Clin Infect Dis.

[25] Ouattara AK, Yameogo P, Traore L, Diarra B, Assih M, Compaore TR (2017). Prevalence, genetic variants and clinical implications of G-6-PD deficiency in Burkina Faso: a systematic review. BMC Med Genet.

[26] Guleria VS, Sharma N, Amitabh S, Nair V (2013). Ceftriaxone-induced hemolysis. Indian J Pharmacol.

[27] Vehapoglu A, Goknar N, Tuna R, Cakir FB (2016). Ceftriaxone-induced hemolytic anemia in a child successfully managed with intravenous immunoglobulin. Turk J Pediatr.

[28] Guinovart C, Sigauque B, Bassat Q, Loscertales MP, Nhampossa T, Acacio S (2022). The epidemiology of severe malaria at Manhica District Hospital, Mozambique: a retrospective analysis of 20 years of malaria admissions surveillance data. Lancet Glob Health.

[29] Marsh K, Forster D, Waruiru C, Mwangi I, Winstanley M, Marsh V (1995). Indicators of life-threatening malaria in African children. N Engl J Med.

[30] Diarra A, Nebie I, Tiono A, Sanon S, Soulama I, Ouedraogo A (2012). Seasonal performance of a malaria rapid diagnosis test at community health clinics in a malaria-hyperendemic region of Burkina Faso. Parasit Vectors.

[31] Tiono AB, Diarra A, Sanon S, Nebie I, Konate AT, Pagnoni F (2013). Low specificity of a malaria rapid diagnostic test during an integrated community case management trial. Infect Dis Ther.

